# How might we interpret quantitative findings in simulation research more meaningfully?

**DOI:** 10.1186/s41077-026-00437-8

**Published:** 2026-04-13

**Authors:** Anders L. Schram

**Affiliations:** MidtSim, Department of Clinical Medicine, Aarhus University, Aarhus, Denmark

**Keywords:** Simulation research, Medical education, Statistical inference, Quantitative interpretation, Statistical significance, Confidence intervals, Effect size, Practical relevance, Confounding, Bias, Measurement

## Abstract

**Background:**

Quantitative findings carry particular authority in simulation research and are often read as decisive evidence that an intervention has—or has not—made a meaningful difference. Statistical significance can function as an interpretive shortcut, providing a ready-made storyline that compresses uncertainty and context. This debate article examines how such readings arise and why they risk exceeding what quantitative results produced in simulation research can legitimately be taken to support.

**Main body:**

The argument distinguishes what common statistical outputs are designed to show from what they are often asked to mean. A p-value describes how incompatible the observed data are with a no-difference model; it cannot by itself demonstrate learning or clarify how and why an observed contrast arose. In simulation research, this gap is often widened when samples are modest and when outcomes reflect behaviour that develops unevenly across clinical settings and over time. Under these conditions, interpretations that hinge on statistical significance alone become fragile. To make these limits explicit, the paper highlights four constraints on inference that clarify what a quantitative contrast can reasonably support: (1) uncertainty around the estimate, made visible through confidence intervals; (2) effect size understood in relation to variability and the conditions under which outcomes are produced; (3) the relevance of observed changes for clinical, organisational or educational work; and (4) the influence of systematic influences including information bias, selection bias, and confounding. A hypothetical emergency department resuscitation programme example illustrates how the same numerical contrast can support different conclusions once these constraints are taken seriously.

**Conclusion:**

Quantitative results remain central to simulation research, but they do not function as verdicts on whether an intervention worked. Their value depends on careful interpretation—treating statistical outputs as inputs to judgment rather than as decisive evidence. Making the constraints on inference explicit supports interpretations that remain proportionate to what the data can sustain and strengthens the development of cumulative knowledge in simulation research.

## Background

Quantitative results carry particular authority in simulation research and, more broadly, in medical education [1–4]. They often arrive in a manuscript with an implicit promise: that numbers can settle whether an intervention “worked.” A p-value below 0.05 is commonly taken as confirmation that a meaningful change has occurred [1, 5–7]. When the value crosses that threshold in the opposite direction, the interpretation often collapses just as quickly, and the intervention is framed as having had little or no influence [1, 7, 8]. This pattern is not unique to medical education, including simulation. The American Statistical Association’s statement on p-values cautioned against treating significance testing as a verdict on effect or importance [5], echoing concerns raised across multiple scientific fields [6]. Spiegelhalter makes the same point from another angle: statistical outputs do not interpret themselves [9]. They show contrasts generated under particular assumptions. What those contrasts mean depends on the conditions that produced them, and on what it is reasonable to infer from those conditions.

In simulation research, those conditions are rarely simple. Many simulation-based interventions and research applications—particularly those concerned with behaviour over time—can be understood as complex interventions embedded in complex adaptive healthcare systems, where outcomes arise through ongoing interactions rather than linear cause–and–effect chains [10]. In such contexts, variability is not noise to be eliminated but a defining feature of the systems that simulation seeks to engage [3, 11, 12]. Learning and adaptation tend to unfold unevenly over time and across organisational settings, making them difficult to reduce to a single outcome metric [13–15].

The consequence is a familiar interpretive tension. A statistically significant result may capture a numerical shift without showing whether anything of practical relevance has changed [8]. A non-significant result may instead reflect imprecision rather than an absence of learning or improvement [8]. When such findings are read through a binary lens, they risk attributing clarity and stability to patterns that are far less certain than the labels suggest. These tensions persist not because the statistics are obscure, but because statistical significance can offer a ready-made storyline. It allows authors to present an intervention as effective—or ineffective—without sustained attention to how the data were produced or what alternative explanations remain plausible [6, 16]. This tendency is reinforced when analytic flexibility remains implicit. In studies where several outcomes are available and analytic decisions evolve during engagement with the data, statistical significance can arise from the analytic trajectory itself rather than reflecting a stable underlying signal. In such situations, the p-value reflects the analytic path taken as much as it reflects the phenomenon under study. This pattern—often described as *p-hacking*—rarely reflects deliberate analytic choices [17]. It echoes the familiar saying that “*if data are interrogated long enough*,* they will appear to confess”*, a reminder that interpretive clarity may arise from analytic choices as much as from underlying change.

This debate article argues for slowing that storyline down by examining how quantitative findings in simulation research and medical education are interpreted. Rather than proposing alternative decision rules, the argument identifies recurring constraints on inference that delimit what quantitative results can reasonably be taken to support. A case example is used later in the article to illustrate how these constraints shape what quantitative findings can reasonably be taken to mean in practice. These constraints include confidence intervals that address uncertainty around estimates, how effect sizes are produced through measurement practices, the practical relevance of observed changes, and the role of systematic influences including information bias, selection bias, and confounding.

### What statistical significance can—and cannot—say

Statistical significance is a familiar touchstone in simulation research [13, 18]. In before–and–after comparisons, the p-value often becomes a proxy for whether a difference is “real” [5–7]. In practice, it functions less as an output and more as an interpretive switch: the finding either matters or it does not. The literature rewards this switch because it produces tidy conclusions and easy summaries.

The problem is that the meaning of a p-value is narrower than the role it is asked to play. A p-value quantifies how incompatible the observed data are with a model that assumes no underlying difference [5]. It does not confirm learning. It does not establish practical importance. It does not explain what produced the contrast [6, 19]. It is shaped by random variation and by the test’s structure, not by the plausibility of a causal account. Table 1 summarises several recurrent ways in which this mismatch plays out in simulation research, where p-values are routinely asked to support claims they cannot sustain. That gap—between what the statistic is and what it is used to do—is where simulation research repeatedly gets pulled off balance [6, 7].

**Table 1:** Common misreadings of *p*-values in simulation research

Misinterpretation	What the *p*-value actually shows	Why it matters in simulation studies
“*p* < 0.05 shows the intervention worked.”	A p-value estimates how surprising the data are under the assumption of no effect. It does not demonstrate that the intervention caused the observed change.	A statistically significant result shows that the data are inconsistent with a no-effect model — not that the intervention itself led to learning or behavioural change.
“*p* ≥ 0.05 means nothing changed.”	A non-significant p-value may reflect low statistical power, measurement noise, or contextual variability — not necessarily the absence of an effect.	Real changes may go undetected in studies with small samples, inconsistent attendance, or imprecise outcome measures — all common features of simulation-based research.
*“The p-value tells us whether the effect is large.”*	The p-value says nothing about the magnitude of an effect. Large effects may yield high p-values (e.g., in small samples), and small effects may be statistically significant.	Practical relevance must be assessed using effect size estimates (e.g., Cohen’s d), not p-values. In simulation, both the size and the contextual meaning of change are critical.
*“A significant result reflects a stable estimate.”*	The p-value reflects results from one dataset. It provides no information about robustness or reproducibility across settings or samples.	Simulation data are shaped by structured variation — assessor-specific variation, scenario differences, team dynamics — which may shift estimates even when results are statistically significant.
*“Significance isolates the effect of the intervention.”*	A p-value does not control for concurrent influences, contextual shifts, or confounding. It only tests the plausibility of the data under a no-effect assumption.	Simulation takes place within dynamic clinical systems. Changes in staffing, workflow, institutional culture, or participant expectations may coincide with the intervention and affect outcomes. Significance cannot disentangle these influences.

In simulation studies, the limitations of significance testing are not abstract. Sample sizes are often modest, exposure is uneven, and outcomes depend on everyday clinical routines, which influence both what is done and what is captured in the data [1, 13, 18, 20]. Under these conditions, statistical significance can appear even when the underlying signal is unstable [21]. The reverse is just as plausible: a non-significant result may conceal a shift that would have been visible under more consistent measurement, or with more information in the dataset [6, 7].

A further complication lies in the assumptions beneath many common tests. Simulation datasets frequently strain assumptions of independence, normality, or equal variance because performance unfolds within teams [22], is judged by a small pool of assessors [23, 24], and produces skewed or bounded distributions [25]. Even when results are reported neatly, the analytic scaffolding can sit on conditions that the data do not meet. The p-value cannot warn you about this. It will still offer its binary temptation [1, 5].

#### Elements that constrain interpretation

**Fig. 1:**
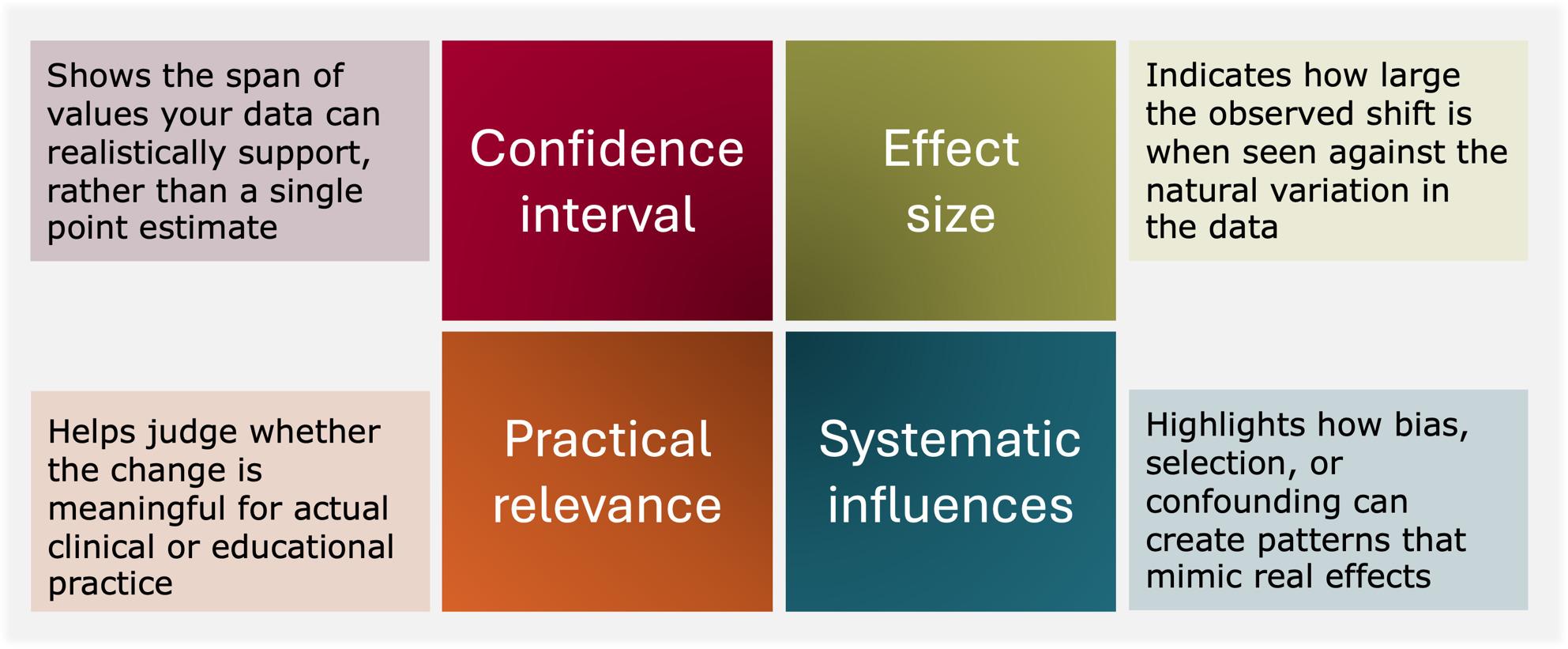
Key elements for interpreting quantitative findings in simulation research

Interpreting quantitative findings in simulation requires attention to features of the data that extend beyond the simple significant/non-significant contrast. Fig. 1 organises this interpretive work around four elements that together constrain what a quantitative result can reasonably support [9, 26]: (1) the uncertainty surrounding the estimate, made visible through confidence intervals; (2) the effect size, understood in relation to variability and the conditions under which measurements are produced; (3) the practical relevance of the observed change for clinical or educational work; and (4) the potential role of systematic influences, where the pattern reflects bias or confounding.

These elements are analytically distinct, and none can stand in for the others. Precision does not compensate for bias. A sizeable effect estimate does not, by itself, indicate educational or clinical importance. Apparent relevance is equally insufficient when the pattern could plausibly reflect selection processes or shifts in measurement rather than a change attributable to the intervention. Taken together, the four elements shift interpretation away from verdicts and toward judgment, encouraging readings that are attentive to uncertainty, context, and the limits of what the data can sustain [9].

#### Confidence intervals and the range they encompass

A confidence interval expresses uncertainty around an estimate. It describes a set of values compatible with the observed data under the model’s assumptions. A 95% interval means that, across repeated studies conducted under identical conditions, intervals constructed in this way would include the true value in 95% of cases [27, 28]. It does not mean there is a 95% probability that the true effect lies inside the interval for this study.

In simulation research, intervals often widen for reasons that are structural rather than incidental. They widen when information is limited, when participation is partial, when scores fluctuate because measurement is sensitive to context, or when outcomes are produced through judgment rather than direct counting—features that recur across simulation designs [29]. Under such conditions, an interval can span values that support sharply different interpretations. Greenland and colleagues highlight a simple point that is often treated as a technical detail: when intervals cover effects with markedly different implications, the dataset cannot sustain a firm conclusion [19]. This is not a failure. It is a signal about what the evidence can and cannot discriminate between.

A pragmatic way to read a confidence interval—without turning it into a new threshold—is to consider what the range of values would imply in practice. If the lower end corresponds to little or no meaningful change, while the upper end would suggest a change that matters for clinical or educational work, the estimate is too imprecise to support strong conclusions. Gelman and Greenland make a related point: the interval communicates limits of inference because it shows how many effects remain plausible given the data [30]. Amrhein and colleagues’ argument for retiring statistical significance pushes in the same direction, emphasising how wide intervals often keep meaningful and trivial effects simultaneously in play [6]. For simulation research, one implication follows from this discussion. Compared with a p-value, confidence intervals often provide more insight into what the data can support, because they make uncertainty and precision visible rather than collapsing interpretation into a binary contrast. Importantly, when an interval includes both very small effects and effects that would matter in practice, the same data can support different decisions about whether a programme should be continued or scaled. In that situation, the result does not justify a clear conclusion that the programme worked.

#### Effect size

An effect size is a statistical estimate intended to indicate how large an observed difference is in practical terms. Rather than simply detecting whether a difference exists, it helps readers judge its potential importance. Effect sizes summarise how large a change appears relative to variation in the data. They are often treated as a step beyond p-values: less binary, more informative, more “about magnitude.” That is true, but only up to a point. Effect sizes are not direct windows into an underlying performance trait. They are compressed summaries of the measurement practices and situational routines that produced the scores. How an effect size should be interpreted depends on the outcome being measured and on how that outcome is produced [28, 31]. Some outcomes change slowly, so even small numerical shifts may be meaningful. Others vary from one observation to the next because scenarios evolve or participation differs across sessions, as is common with team-based ratings or locally adapted scenarios. In those situations, a large effect size may reflect features of the measurement process rather than a stable change in learning.

Much of the variation that feeds effect sizes is patterned rather than random. It can reflect assessor drift, local adaptation of scenarios, changing clinical tempo, and participation shaped by workload. When these dynamics are folded into a standardised contrast, the resulting number blends potential learning with contextual production effects. Treating the effect size as direct evidence of impact risks mistaking “*what the system produced*” for “*what the intervention caused*” [32, 33]. 

Exposure matters as well. When only some staff attend training, or when attendance clusters among particular professional groups or shifts, the effect size may reflect who was measured rather than what changed [34]. This uneven “dose” is not a nuisance variable; it is an intrinsic feature of clinically embedded simulation, and it complicates claims that the effect size represents a unified programme effect. Because analytic choices shape how these patterns appear, specifying planned analyses in advance—typically through formal registration of the study or trial protocol—can reduce the scope for post-hoc emphasis. Such preregistration does not eliminate judgment, but it makes the analytic intentions explicit before results are known, allowing readers to see which decisions were planned and which were actually conducted during the analysis [6].

#### Practical relevance

Statistical outputs are sometimes read as if they can tell us whether a simulation intervention matters in clinical work. They cannot. A dataset can show a convincing contrast, while practice remains largely unchanged when pressure rises [35, 36]. The opposite is also plausible: a change that falls short of statistical significance may still reflect adjustments that clinicians recognise as important when they describe coordination and decision-making in real cases [32].

Practical and educational relevance rests on a different question than statistical detection: *would the change be noticeable in everyday work?* An effect becomes meaningful when it corresponds to something that clinicians and educators would plausibly recognise outside the scenario—less hesitation as the pace increases, smoother transitions between tasks, fewer moments where the team stalls because no one can see what comes next [37, 38]. If a measured difference does not map onto anything that would matter in practice, its educational value remains uncertain even when the numerical contrast looks strong.

Multiplicity widens this problem. Many simulation studies include several quantitative outcomes from different sources—behavioural ratings, checklists, timing measures, self-reports [18, 39, 40]. This can be appropriate, but it also expands the interpretive space. When a single favourable metric becomes the centre of attention, it can stabilise an “effect” narrative even if the broader pattern is inconsistent or difficult to read [41]. This concern aligns with wider critiques about flexible analyses and fragile single-indicator claims [42].

Simulation programmes can also influence aspects of practice that sit outside the chosen outcome [10]. Teams may describe more predictable routines before demanding tasks, fewer unspoken assumptions, or a steadier sense of how to coordinate under pressure [43]. These developments rarely appear in the primary metric, yet they often capture what clinicians value. They do not override quantitative findings, but they caution against treating any single effect size as a complete account of impact [10].

#### Systematic influences

Systematic influences arise when the conditions under which data are produced introduce patterned distortions into estimates [22, 44]. In simulation research, these influences are common and can make an interpretation feel more secure than the evidence allows, especially when the storyline is driven by a single number [45]. Often, the source of these problems lies earlier than the analysis itself. Design and documentation decisions are frequently made while a simulation programme is being implemented under everyday operational pressures. When programmes begin as quality improvement initiatives and are later analysed as research, measures are often chosen for practical reasons rather than analytic clarity [46]. As a result, it may be unclear why particular outcomes were selected, how consistently they were applied, or what else changed during the same period. Research on the quality improvement–research boundary describes this as a common pattern: choices made to support operations end up shaping what can later be measured, and thereby what can be inferred from the data [46]. These influences often become visible through three recurring forms of systematic misrepresentations in simulation research: information bias, selection bias, and confounding.

##### Information bias

Information bias occurs when the measurement process systematically shapes the result. It enters through how outcomes are captured or interpreted, steering the estimate away from underlying behaviour [22]. In simulation research, performance ratings illustrate this risk. Scoring depends on how assessors interpret what they see. When assessors know the participants or recognise local ways of working, their impressions can influence how criteria are applied [32, 45]. The resulting scores may blend performance with expectation. Such influences cannot be removed entirely, but they can be made more visible when studies describe how ratings are produced and when researchers consider how local norms shape judgment over time [32]. Several practical steps during implementation may help limit this form of misrepresentation in the data. Piloting simulation scenarios can identify threats to data capture before a formal study begins [47, 48]. Training and standardisation of simulated patients or assessors, alongside attention to inter-rater agreement, can strengthen the credibility of performance ratings [47, 49]. Where feasible, incorporating more objective outcome measures may further reduce interpretive variation [50]. These strategies do not eliminate measurement error, but they can help ensure that observed variation more closely reflects performance rather than inconsistencies in how it is recorded [49].

##### Selection bias

Selection bias emerges when the individuals who provide data differ systematically from the group the study aims to describe. It concerns who ends up being measured [22, 51]. Questionnaire data provide a clear example. Staff who feel aligned with the simulation programme or are comfortable with teamwork may be more likely to respond, while others decline. If the dataset reflects this subgroup, the results describe their experience rather than the department as a whole [51, 52]. Some of this imbalance can be tempered through transparent reporting of response patterns and by examining whether those who contribute data differ meaningfully from those who do not. Still, the underlying uncertainty always remains part of the interpretive terrain [22].

##### Confounding

Confounding occurs when factors outside the intervention influence the outcome in ways that resemble an intervention effect. Some threats to inference can be addressed at the design stage. Strategies such as randomisation, crossover designs, and protocol standardisation may reduce systematic imbalance [53]. Yet when complex interventions are studied within dynamic clinical systems, design features rarely resolve these concerns, because exposures and contextual conditions continue to evolve over time [10, 48]. This remains a persistent challenge in simulation research, where studies often follow participants across extended periods [54]. Researchers, therefore, attempt to account for potential confounders by measuring background characteristics and adjusting for them. In many simulation studies, such adjustments help stabilise estimates when factors such as age or profession shape learning or performance [18]. Some designs also incorporate a contemporaneous control group, allowing broader contextual shifts to become visible [48]. However, these strategies address only what is measured. Many simulation programmes unfold in settings where conditions shift in ways that rarely appear in the dataset [10]. Work may be shaped by who is available on a given day or by new pressures that alter how tasks are carried out. Even personal circumstances will likely affect the attention and energy people bring to their shifts, including factors we rarely consider, such as family responsibilities or major life events. These developments can influence findings at post measurements and follow-up without having any connection to the intervention itself [32].

Approaches that integrate qualitative inquiry with quantitative analysis cannot eliminate these influences, but they can illuminate the mechanisms and contextual developments that shape outcomes. Qualitative insights of everyday teamwork and adaptation can help clarify whether a change reflects the intervention’s influence or stems from developments elsewhere in the system [39, 55]. This does not resolve confounding, but it supports a more grounded interpretation of quantitative signals that could otherwise be read in several ways [48].

## Example: what the numbers allow us to say

To anchor these issues, consider a plausible scenario. An Emergency Department becomes concerned about how teams manage resuscitation cases, particularly when coordination falters or key actions are delayed. The department introduces a simulation-based training programme intended to strengthen resuscitation performance across the workforce. The programme runs for twelve months, so staff can participate at least once. Sessions occur intermittently, with participation shaped by workload—an arrangement that mirrors how hospital-based simulation often operates [56, 57]. The programme is designed to fit clinical reality rather than to control it. The primary outcome is the average Team Emergency Assessment Measure (TEAM) score at baseline and again after twelve months. TEAM yields a numerical score based on leadership, coordination, and task management, alongside a global rating [58]. Ratings are carried out by local assessors. On the surface, the evaluation looks straightforward: a recognised need, a targeted intervention, and a pre–post comparison. Many manuscripts stop there. But once the contrast is read through uncertainty, variability, practical relevance, and systematic influences, the interpretive terrain changes [48]. Table 2 illustrates how confidence intervals, effect size, practical relevance, and the major forms of systematic influences shape what the results appear to show—and what they can legitimately support. The same result can support different conclusions. One interpretation may take the observed increase as sufficient reason to continue or expand the programme. Another may focus on the uncertainty around the estimate and how uneven participation and local conditions shaped the results, leading to a more cautious decision. The difference lies not in the data, but in what is taken to be enough evidence to act.

**Table 2:** Elements that inform interpretation and how they apply in the hypothetical resuscitation scenario

Element	What it draws attention to	Interpretation in resuscitation example
Confidence interval	The range of values compatible with the observed data, reflecting the precision of the estimate. A wide interval signals substantial uncertainty about the true effect.	If the TEAM score increases but the confidence interval is wide, many values remain plausible—including ones with no meaningful change. This constrains how confidently the observed difference can be attributed to improved team performance.
Effect size	The magnitude of the observed difference relative to variability in the data. It quantifies contrast but does not determine its importance.	A larger effect size may support the interpretation that teams move more reliably through critical tasks. A small effect size suggests a modest difference but does not by itself imply that the change lacks practical relevance—it must be judged in context.
Practical relevance	Whether the observed pattern corresponds to changes that make a difference in real work. Statistical significance alone is insufficient.	A minor score shift might reflect more fluid transitions between key actions, which teams would notice in practice. Conversely, a larger shift might concern scenario-specific behaviours with little bearing on actual clinical work. What matters is *what* changes, and whether it matters to performance.
Information bias	Systematic influences arising from how outcomes are captured or interpreted. This includes observer effects, misclassification, or rater expectations.	If local assessors are familiar with participating teams, their expectations may influence scoring—resulting in apparent improvement that reflects how performance is judged rather than how resuscitations are actually managed. This affects the credibility of any link to learning.
Selection bias	Systematic differences between those who provide data and those the study aims to represent. Participation is not always random.	If the staff assessed after twelve months are disproportionately confident in simulation, the rise in TEAM score may reflect their prior attributes, not a general departmental change. This limits generalisability.
Confounding	External influences that affect outcomes and may mimic or obscure intervention effects. They reduce internal validity if not addressed.	Changes in staffing, workflows, or parallel initiatives may influence performance independently of the simulation programme. Without considering these, we cannot isolate how much of the improvement stems from the training itself — and over extended periods, such isolation is rarely possible when studying complex team behaviours in clinical settings.

The point is not that such studies are futile. It is that a single numerical contrast cannot bear the interpretive load that simulation research often places on it. The more clinically authentic the setting, the more the data are entangled with the system that produced them [59]. That entanglement does not negate inference, but it forces the inference to be stated more carefully.

Taken together, the issues discussed above point to a broader interpretive responsibility in simulation research. Quantitative findings do not speak for themselves; what they seem to show depends on how uncertainty is handled and on whether alternative explanations are taken seriously when results are analyzed and written up. These questions are not confined to methodological debate. They arise as studies take shape and as researchers work with their data and decide how to communicate what the results are taken to mean. For researchers working across both research and quality improvement, this invites reflection on how quantitative findings are produced and framed in their own projects, and on how claims about change are expressed in light of what the data can reasonably support.

## Conclusions

Simulation research frequently relies on quantitative results, yet those results are often interpreted with more decisiveness than the evidence supports. This article does not argue against quantitative work; rather, it calls for interpretations that remain anchored in what the data can reasonably sustain. Statistical tools reveal patterns, but they do not determine whether an intervention altered anything that matters in practice. Quantitative findings, therefore, gain their value through the judgments that accompany them — through analytic transparency, theoretical positioning, and careful consideration of alternative explanations when conclusions are drawn.

Strengthening simulation research depends not only on methodological refinement but on interpretive practices that remain proportionate to what the data can support. Such discipline allows claims about change to travel more responsibly across settings and supports the development of knowledge that builds cumulatively rather than resting on isolated findings. 

Many studies conducted in real-world educational and clinical settings will, for understandable reasons, include sources of bias that are difficult to eliminate entirely. The issue is therefore not the presence of such limitations in itself, but whether their implications are recognised and considered when interpreting findings. Careful attention to these risks allows conclusions to remain proportionate to what the evidence can support and supports more transparent knowledge development.

## Data Availability

Data sharing is not applicable to this article as no datasets were generated or analysed during the current study.

## Abbreviations

TEAM: Team Emergency Assessment Measure
